# Bilateral Intravitreal Ranibizumab Injection and Panretinal Photocoagulation in a 16-Year-Old Girl with Severe Vaso-Occlusive Lupus Retinopathy

**DOI:** 10.1155/2013/817186

**Published:** 2013-12-09

**Authors:** Hasan Can Doruk, Pinar Cetin, Fatos Onen, A. Osman Saatci

**Affiliations:** ^1^Department of Ophthalmology, Dokuz Eylul University, Mithatpasa Cad, 35340 Izmir, Turkey; ^2^Department of Rheumatology, Dokuz Eylul University, Mithatpasa Cad, 35340 Izmir, Turkey

## Abstract

A 16-year-old girl with fever of unknown origin and bilateral vaso-occlusive retinopathy with retinal neovascularization, preretinal hemorrhage, and serous macular detachment was treated with single bilateral 0.5 mg intravitreal ranibizumab injection prior to aggressive PRP with success. No systemic steroids or immunosuppressive therapy was employed at that time. She received the diagnosis of “systemic lupus erythematosus” five years after this episode with further systemic symptoms. In certain cases with vaso-occlusive type of lupus retinopathy, anti-VEGF agents may be administered in addition to panretinal photocoagulation to achieve better visual and anatomic outcome.

## 1. Introduction

Systemic lupus erythematosus (SLE) is a multisystem autoimmune disorder of unknown aetiology associated with the presence of circulating autoantibodies to one or more components of cell nuclei and immune-complex mediated tissue destruction [1].

The retina is a common site of ocular involvement in patients with SLE [2–4]. Lupus retinopathy is mostly characterized with microangiopathy associated with arterial occlusion and it presents with cotton-wool exudates and intraretinal hemorrhages. The prevalence of lupus retinopathy ranges from 3% in an outpatient population with mild to absent disease to 29% among patients with active disease [5]. Most of the time visual prognosis is excellent. However, when a vaso-occlusive retinopathy occurs, the visual outcome may be severely compromised. Vaso-occlusive retinopathy is a much rarer form of retinal involvement and associated with widespread retinal capillary nonperfusion, multiple branch retinal artery occlusion, ocular neovascularisation, vitreous hemorrhage, and tractional retinal detachment [6–8]. On the other hand, retinal involvement was rarely seen in pediatric subgroup of patients. In a group of 52 consecutive children with SLE (mean age was 11.3 years), five patients had (four unilateral, one bilateral) retinal vascular changes [9]. Few patients under 18 years of age with vaso-occlusive type of retinopathy were described in previous reports [10].

In this case report, we described a 16-year-old girl with bilateral severe vaso-occlusive retinopathy who was treated successfully with combined treatment of single ranibizumab injection followed by panretinal photocoagulation without any systemic treatment as there was no established systemic diagnosis at that time and the patient was diagnosed as having SLE five years after the initial episode.

## 2. Case Report

**Figure 1:**
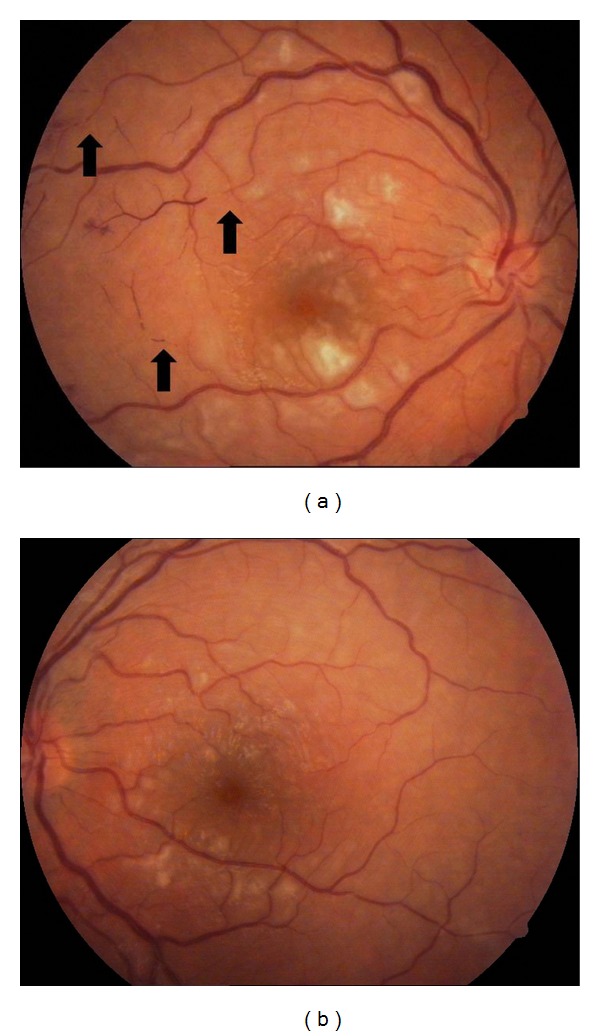
(a) Right eye, color fundus picture depicting widespread cotton-wool spots and arteriolar occlusion (Black arrows). (b) Left eye, color fundus picture showing multiple cotton-wool spots.

**Figure 2:**
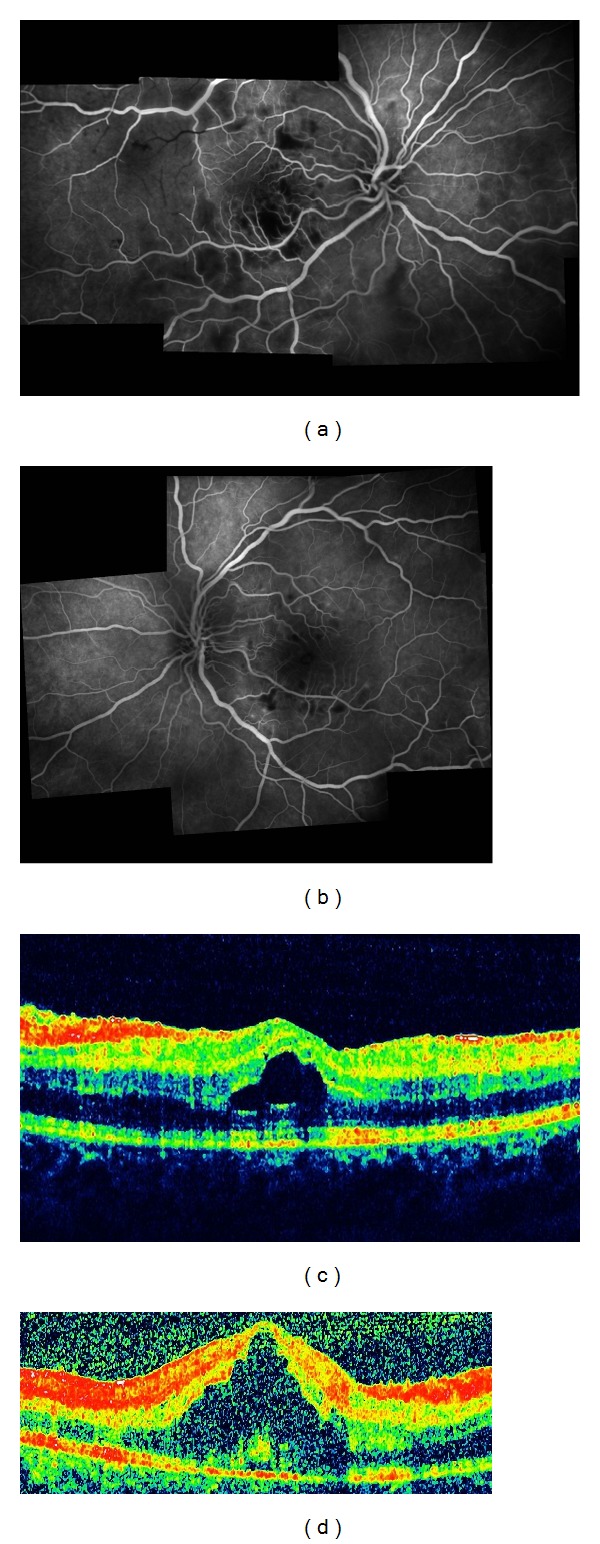
(a) Right eye, composite fluorescein angiogram demonstrating extensive capillary nonperfusion and arteriolar occlusion. (b) Left eye, composite fluorescein angiogram showing capillary dropout areas at the posterior pole. (c) Right eye, OCT picture demonstrating serous macular detachment. (d) Left eye, OCT picture exhibiting serous macular detachment.

**Figure 3:**
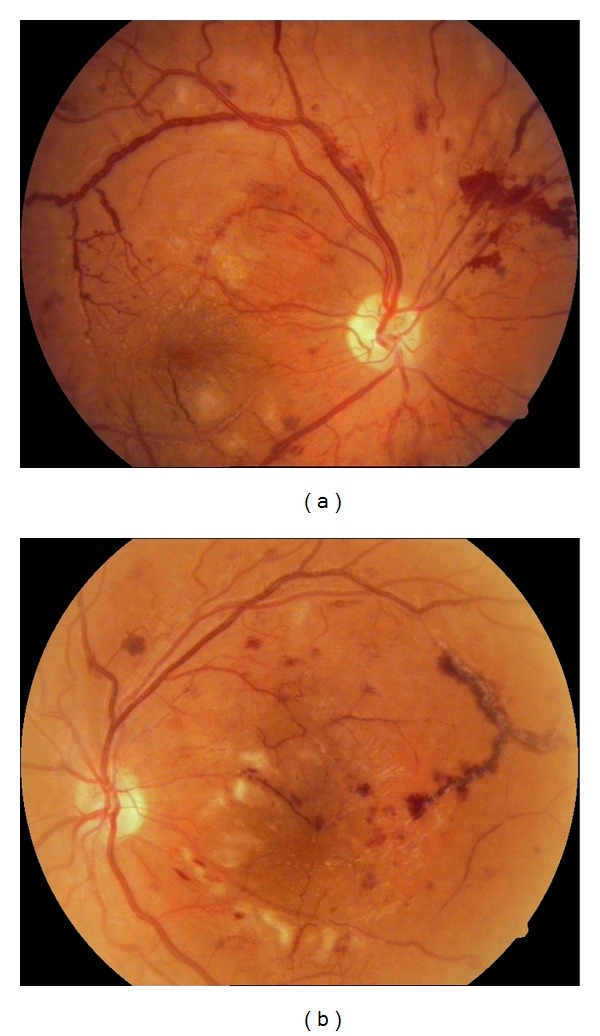
(a) Right eye, color fundus picture taken 45 days after the first presentation showing venous beading, soft-exudates, preretinal hemorrhage and retinal neovascularization. (b) Left eye, color fundus picture showing progression of the vaso-occlusive retinopathy characterized with occlusive vasculitis, widespread cotton-wool exudates and retinal hemorrhages.

A-16-year-old girl with no past medical history was hospitalized with fever of unknown origin and blurry vision OU. On ophthalmological examination, her best corrected visual acuity was 3/10 OD and 4/10 OS. Intraocular pressures and slit-lamp examination were normal. Fundus examination revealed widespread cotton-wool exudates (Figures 1(a) and 1(b)). Fluorescein angiogram demonstrated widespread 360° severe capillary nonperfusion and arteriolar occlusion (Figures 2(a) and 2(b)). Optical coherence tomography exhibited serous macular detachment OU (Figures 2(c) and 2(d)). Her physical examination revealed no systemic findings except the fever. Except leukopenia, mild anemia, elevated acute phase proteins, and hepatic markers, all other systemic evaluations including autoimmune markers (anti-nuclear antibody (ANA), anti-dsDNA, anti-RO, lupus anticoagulant, and anticardiolipin antibodies) infection markers, genetic mutation analysis for FMF, bone marrow biopsy, and cranial magnetic resonance imaging (MRI) were normal. Blood cultures were also negative. As urine culture was positive for enterococcus, she received intravenous ampicillin/sulbactam for five days and then the therapy was switched to oral amoxicilline. Fever subsided during the course. However, vaso-occlusive disease progressed and preretinal hemorrhage and neovascularisation were observed in OU after 45 days (Figures 3(a) and 3(b)).

**Figure 4:**
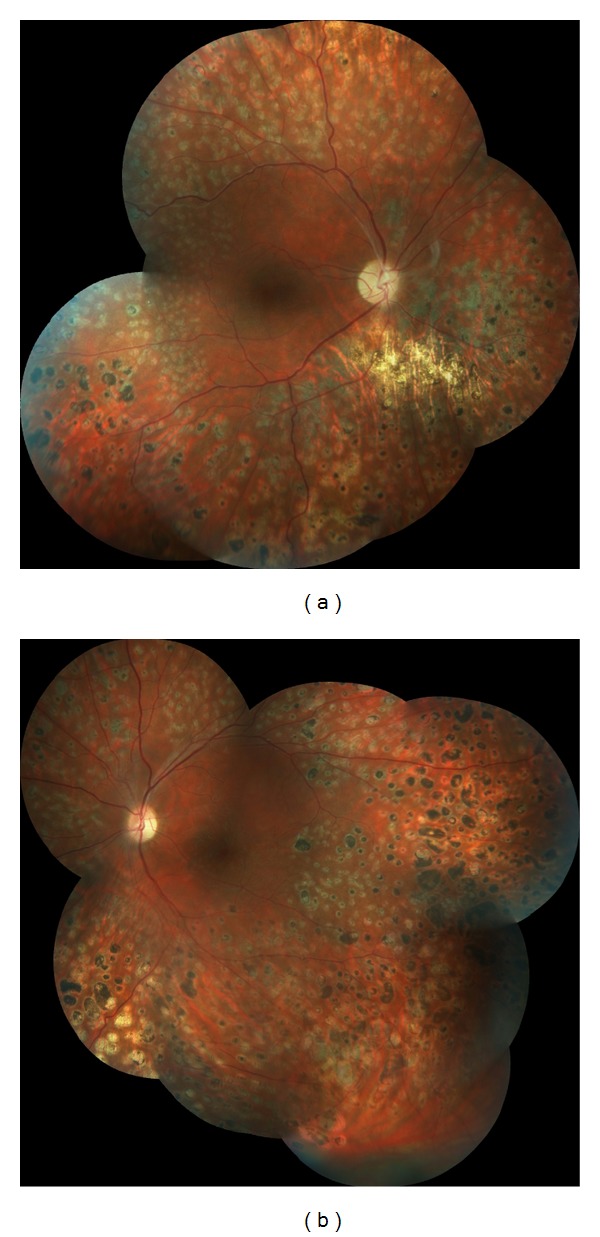
Composite color fundus pictures showing no sign of disease activity with the full laser scars of panretinal photocoagulation ((a) right eye, (b) left eye).

After explaining the visual prognosis and possible poor outcome, we proceeded with bilateral 0.5 mg intravitreal ranibizumab injection to prevent macular edema worsening following the PRP. Then, PRP was administered in two sessions a week apart 3 days after the injection OU. The retinopathy becomes stabilised and the vision increased to 8/10 OU. She attended her follow-up ophthalmological examinations regularly with no sign of activity OU (Figures 4(a) and 4(b)). Five years after the initial episode, she was admitted to rheumatology clinic for symmetrical polyarthritis of both hands and fever. She also had photosensitivity, macular rash, and alopecia. This time, the laboratory evaluation revealed leukopenia, a positive ANA titer of 1 : 1000 with granular pattern, positive anti-Ro, and anti-RNP antibodies and the diagnosis was systemic lupus erythematosus (SLE). Thereby, hydroxychloroquine treatment was commenced.

## 3. Discussion

Several aspects of present 16-year-old girl with vaso-occlusive retinopathy were noteworthy: (1) time lapse of five years between the initial presentation and the diagnosis of SLE, (2) concomitant use of ranibizumab and PRP for the treatment of severe bilateral vaso-occlusive retinopathy, and (3) the success of local therapy without any concomitant systemic treatment as the diagnosis of SLE could be established later.

Panretinal photocoagulation should be employed in patients with SLE whenever vasooclusive retinopathy was detected. In a review by Au and O'day [1] in 2004, authors suggested that the process of capillary nonperfusion was generally irreversible in eyes with severe vaso-occlusive retinopathy and disc or retinal neovascularisation almost always ensues. Regression of neovascular process could only be achieved in 55% of eyes with aggressive PRP.

Very recently anti-VEGF agents mostly bevacizumab were also employed in limited number of patients [11, 12]. Lee et al. [12] reported two adult patients with bilateral severe vaso-occlusive retinopathy. In their first case, unilateral NVD developed despite ongoing systemic therapy with methotraxate (10 mg/day) and oral prednisone. The patient was treated with 1.25 mg intravitreal bevacizumab injection and concomitant PRP with full regression of NVD. However, in the second case despite initial systemic 1 g/day pulse steroid therapy, cyclosporine 250 mg/day, aspirin treatment and bilateral intravitreal 1.25 mg bevacizumab and subtenon triamcinolone acetonide injection retinal vascular obstruction progressed and monthly intravenous cyclophosphamide 500 mg/m^2^ was commenced. While PRP was adequate for the control of NVE in the right eye, fellow eye developed vitreous hemorrhage and tractional RD and underwent vitrectomy. Jeon and Lee [11] injected 1.25 mg intravitreal bevacizumab for an unilateral vaso-occlusive lupus retinopathy in a 22-year-old female in addition to scattered laser photocoagulation for widespread retinal ischemia and macular edema. However, one day after the injection, visual acuity decreased to counting fingers and fluorescein angiography revealed extension of capillary nonperfusion and authors urged caution when employing intravitreal bevacizumab in eyes with vaso-occlusive retinopathy and SLE.

We believe that intravitreal ranibizumab injection given prior to PRP affected the visual and anatomic outcome positively by alleviating the serous macular detachment without any systemic treatment and in selected cases with vaso-occlusive type of lupus retinopathy, combined treatment with anti-VEGF agents and PRP may yield better outcome.
